# Using Four Machine Learning Methods to Analyze the Association Between Polycyclic Aromatic Hydrocarbons and Visual Impairment in American Adults: Evidence from NHANES

**DOI:** 10.3390/toxics12110789

**Published:** 2024-10-29

**Authors:** Xiaowei Zang, Wei Zhou, Hengguo Zhang, Xiaodong Zang

**Affiliations:** 1 College of Safety Science and Engineering, Nanjing Tech University, Nanjing 211816, China; nanozang@njtech.edu.cn; 2 Department of Nephrology, The Children’s Hospital, Zhejiang University School of Medicine, National Clinical Research Center for Child Health, Hangzhou 310052, China; dracozhou@zju.edu.cn; 3 Key Laboratory of Oral Diseases Research of Anhui Province, College & Hospital of Stomatology, Anhui Medical University, Hefei 230032, China; 4 Department of Dental Implantology, College & Hospital of Stomatology, Anhui Medical University, Hefei 230032, China; 5 Department of Pediatrics, The First Affiliated Hospital of USTC, Division of Life Sciences and Medicine, University of Science and Technology of China, Hefei 230001, China; 6 Institute of Public Health Sciences, Division of Life Sciences and Medicine, University of Science and Technology of China, Hefei 230027, China

**Keywords:** PAHs, visual impairment, weighted quantile sum regression, Bayesian kernel machine regression, 2-fluorene, alkaline phosphatase

## Abstract

The causes of visual impairment are complex and may be influenced by exposure to environmental pollutants. Using data from the 2003–2004 National Health and Nutrition Examination Survey (NHANES), we examined the association between exposure to ten polycyclic aromatic hydrocarbons (PAHs) and vision problems in 1149 U.S. adults. We employed various supervised learning methods, including variable selection techniques such as Lasso and elastic net, weighted quantile sum regression (WQS), and Bayesian kernel machine regression (BKMR), to assess the association between PAHs and the occurrence of visual impairments. The mediation effects between urinary 2-fluorene and inflammation were evaluated using mediation analysis. Both the lasso and elastic net models consistently identified two specific PAH congeners, 2-fluorene and 1-phenanthrene, as significant predictors. The WQS regression revealed a positive relationship between the PAH mixture and visual impairment, with notable contributions from urinary 2-fluorene (weight = 0.39) and 9-fluorene (weight = 0.21). BKMR analysis indicated that the likelihood of visual impairment increases with higher PAH exposure, showing a general upward trend. This trend also revealed a positive association between visual impairment and exposure to four specific PAH metabolites, including 2-fluorene. A significant mediation effect was observed for alkaline phosphatase (*p* = 0.03), with a proportion mediated of 10.48%. Our findings suggest a significant association between PAHs and visual impairment, with multiple statistical models consistently emphasizing the crucial role of 2-fluorene exposure. This study highlights the importance of considering environmental pollutants as significant contributors to visual health outcomes, providing insights for preventing visual impairment.

## 1. Introduction

Visual impairment (VI), a prevalent global disability, is categorized into blindness and low vision. In the United States, blindness is defined as having a best-corrected vision of 20/200 or worse, while the World Health Organization (WHO) sets this threshold at 20/400 or worse. Low vision is defined as vision less than 20/40 in the U.S. and under 20/60 according to WHO standards [1]. This condition is a major public health issue due to its broad impact and the significant reduction in quality of life it causes for affected individuals [2,3,4]. The number of people globally affected by moderate to severe VI is projected to increase from 217 million in 2015 to 588 million by 2050 [3]. In the United States, individuals often rate loss of vision as a greater concern than loss of memory, hearing, or speech, placing it among the top four most feared adverse health outcomes [5]. 

Similarly, VI has become a growing concern in China [6]. In 2019, the age-standardized prevalence of moderate and severe VI in China was 2.57% and 0.25%, respectively, with 0.48% of the population affected by blindness. Between 1990 and 2019, the number of individuals with moderate VI rose by more than 133%, and those with severe impairment by 147%, mainly due to population aging [6]. This trend reflects the broader situation in Asia, which is home to more than half of the world’s population [7]. Over the same period, the burden of blindness and VI in Asia surged, with disability-adjusted life years (DALYs) from vision loss increasing by 90.1% and the prevalence of blindness and VI rising by 116%. In 2019, Asia recorded 15.84 million DALYs and 506.71 million cases of VI, primarily due to cataracts, refractive errors, and near VI [7]. South Asia experienced the highest disease burden, particularly affecting women, the elderly, and those with a lower socioeconomic status [7]. Like in the U.S., tackling VI is a major public health priority in China and across Asia. Although our understanding of the epidemiology of VI is improving, its precise etiology remains unclear.

Previous studies have explored the association between chemical exposure and VI [8,9,10]. For instance, Baumann et al. utilized zebrafish as a model organism to demonstrate that embryonic exposure to tetrabromobisphenol A caused significant pathological alterations in ocular development. The changes involved reductions in the size of the eyes, diameter of retinal pigment epithelium cells, and overall pigmentation, resulting in impaired visual functions in the larvae [10]. Similarly, Chen et al. reported that exposure to ambient gaseous pollutants such as SO_2_ and CO is correlated with increased VI in children [11]. Furthermore, PAHs, which result from the inadequate burning of organic compounds such as petroleum, natural gas, coal, and tobacco, infiltrate human surroundings through multiple pathways [12]. Known for their persistence and bioaccumulative potential, PAHs are closely linked to chronic health effects through mechanisms such as oxidative stress, inflammation, DNA methylation, and glutamate signaling, which notably impact the nervous system [13,14]. Systematic reviews and meta-analyses involving 428 studies have demonstrated that prenatal exposure to PAHs significantly impacts children’s intelligence, psychological development, verbal IQ, memory, and behaviors [15,16,17]. 

Recent research has further associated exposure to PAHs with compromised cognitive function and increased vulnerability to neurodegenerative disorders [18]. Due to the high lipid solubility of PAHs, these chemicals are readily absorbed through the digestive system of mammals. Once inside an organism, PAHs undergo metabolism to form hydroxylated and glucuronidated derivatives, potentially triggering a cascade of oxidative stress responses that may affect the visual nervous system [19]. Emerging evidence suggests that PAHs may induce systemic inflammation, contributing to various health outcomes, including neurotoxicity, angiogenesis, and metabolic syndrome [20,21,22]. Therefore, inflammatory markers like white blood cell count (WBC), lymphocyte count (LYM), albumin (ALB), alkaline phosphatase (ALT), iron, and C-reactive protein (CRP) could serve as indicators of inflammation and offer insights into the potential mechanisms underlying the association between PAH exposure and VI.

However, there is currently no direct evidence connecting PAH exposure with specific VI. With the prevalent exposure and potential health consequences in mind, Understanding the association between PAHs and VI could hold significance for public health. Nonetheless, previous studies focusing on PAH exposure and its effects on health outcomes have largely concentrated on individual or similar exposures, neglecting the complex interactions that come with exposure to multiple PAHs simultaneously. Additionally, conventional research approaches often struggle to select highly interconnected environmental pollutants and lack sensitivity in evaluations [23,24]. Thus, in this work, we utilized machine learning techniques to gain insights into the effects of PAH exposure on VI in adults. These techniques, including supervised methods, may enable us to evaluate the collective impacts of PAH exposure by taking into account potential nonlinear relationships and interactions.

## 2. Methods

### 2.1. Study Population

**Figure 1:**
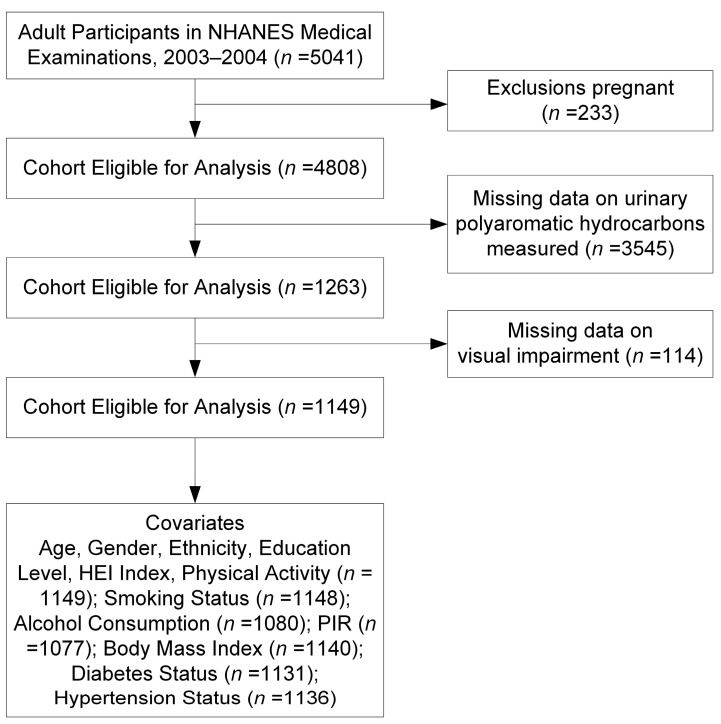
Flowchart of the study population.

The National Center for Health Statistics (NCHS) conducts the NHANES, an extensive cross-sectional study that assesses the health, nutritional status, and lifestyle preferences of the American civilian population. This survey utilizes a sophisticated, stratified, multistage probability sampling approach to reliably choose a sample that represents the population during each two-year research period. Specifically, during the 2003–2004 cycles, 5041 adults were interviewed. From this cohort, we excluded pregnant individuals (*n* = 233), those without necessary environmental chemical data (*n* = 3545), and participants missing information on VI (*n* = 114). As a result, the final cohort for our analysis comprised 1149 participants (Figure 1). The approved NHANES protocol was reviewed by the Research Ethics Review Board (ERB) at the National Center for Health Statistics, an entity within the Centers for Disease Control and Prevention. Public access to documentation on the ERB’s authorization and the data user agreement is available at the following URL: https://www.cdc.gov/nchs/nhanes/irba98.htm (accessed 1 May 2024).

### 2.2. Analysis of Urinary PAHs in NHANES 2003–2004

After processing and storage, the urine samples were analyzed by the Division of Environmental Health Laboratory Sciences at the National Center, under the Centers for Disease Control and Prevention. The analytical procedure included enzymatic urine decomposition, followed by extraction, derivatization, and analysis via capillary gas chromatography coupled with high-resolution mass spectrometry. Isotope dilution with carbon-13-labeled internal standards was utilized, and ion monitoring was conducted to quantify the analytes and their labeled counterparts. The ratios of these ions served as a basis for data interpretation, indicating the level of PAH exposure in the body. Urine samples from over 90% of participants contained ten types of PAHs, including 1-naphthol, 2-naphthol, 3-fluorene, 2-fluorene, 3-phenanthrene, 1-phenanthrene, 2-phenanthrene, 1-pyrene, 9-fluorene, and 4-phenanthrene. Values below the detection limit were adjusted by dividing by the square root of two, and urinary PAH levels were normalized to creatinine concentrations to account for dilution. Detailed information on detection rates for these PAHs is provided in Supplementary Table S1. The NHANES offers a comprehensive explanation of its measurement techniques in the laboratory methods section of its online platform.

### 2.3. Visual Acuity Assessment Method

During the 2003–2004 NHANES, vision examinations were conducted to assess visual acuity in participants, with a particular focus on those whose vision was 20/30 or worse. Individuals who were completely blind, unable to see the light with both eyes open, or had severe eye infections causing purulent discharge with redness and inflammation were excluded from the vision exam. The evaluation began by first removing any corrective lenses. Objective refraction was then quantitatively assessed using an autorefractor (ARK-760; Nidek Co., Ltd., Tokyo, Japan) at a mobile examination facility. In all the statistical analyses, the visual acuity of the better eye was utilized. Parallel results were obtained when the visual acuity of the poorer eye was analyzed. Visual acuity measurements were recorded using discrete Snellen scores, which constrains the analysis to noncontinuous data on visual acuity. Visual acuity was classified into two categories: (i) no VI for visual acuity ≥ 20/40 and (ii) VI for visual acuity < 20/40 [25]. Additionally, for individuals aged 50 and over, their near vision was tested, and their reading distances were recorded. Participants with incomplete data for both eyes or whose visual acuity was consistently worse than 20/40 without autorefraction were excluded from the analysis. This approach ensured consistency in data processing and accuracy in analytical outcomes. For detailed information about the vision examination protocol used during the NHANES, please visit https://wwwn.cdc.gov/Nchs/Nhanes/2003–2004/VIX_C.htm (accessed 1 May 2024).

### 2.4. Detection of Inflammatory Parameters

The inflammatory markers assessed included WBC, LYM, ALB, ALP, iron, and CRP. The complete blood count measures total white blood cells and classifies subtypes such as LYM, supporting overall health assessments and the detection of infections. The DcX800 system uses multiple analytical methods for assessing biochemical markers in serum or plasma. ALB is measured via a bichromatic digital endpoint method where it binds with Bromcresol Purple, indicating concentration levels through changes in absorbance at 600 nm. ALP activity was determined by a kinetic rate method using 2-amino-2-methyl-1-propanol buffer, and the changes in absorbance at 410 nm were tracked as ALP hydrolyzes p-nitrophenylphosphate. Iron levels are quantified using a timed-endpoint method, where iron released from transferring is reduced and then complexed with FerroZine Iron Reagent, with absorbance changes at 560 nm reflecting concentration. CRP was quantified with latex-enhanced nephelometry. All the data were log-transformed to base-10 to address the skewness in the distributions for further statistical analysis.

### 2.5. Covariates

A variety of covariates were taken into consideration to address potential confounders. Sociodemographic characteristics included age, gender, racial/ethnic background (Hispanic, non-Hispanic White, non-Hispanic Black, and other racial groups), educational level (less than a high school diploma, high school diploma, some college or higher), and income (poverty index ratio, PIR). The behavioral risk factors that were analyzed included smoking status (never, current, or former smoker), alcohol consumption (none, moderate, or heavy), body mass index (BMI) (normal/underweight) (<25 kg/m^2^), overweight status (25–30 kg/m^2^), obesity status (≥30 kg/m^2^), and physical activity level (low, adequate, or high according to established guidelines) [26,27]. Diet quality was evaluated using the Healthy Eating Index (HEI)-2015 [28], which assesses the intake of 12 key dietary elements, including fruits, vegetables, grains, proteins, dairy, fatty acids, sodium, and discretionary calories. Chronic conditions such as diabetes and hypertension were determined using fasting glucose levels (≥126 mg/dL or self-reported diagnosis) and resting blood pressure (≥140/90 mmHg or self-reported diagnosis), respectively. The covariates mentioned above were selected based on previous literature, as they have been reported to be associated with both the exposure and the outcomes, and thus were considered potential confounders. For further information on these methodologies, please refer to our earlier publications [29,30,31,32].

### 2.6. Statistical Analysis

Descriptive statistics were reported in their raw form, with no transformations applied. For continuous variables, the data are presented as the mean ± standard deviation for normally distributed data and as the median (interquartile range) for non-normally distributed data. Categorical variables are displayed as frequencies or percentages. To investigate the relationships among the urinary PAH metabolites, Spearman’s correlation analysis was utilized. Total PAHs were summed as ∑PAHs and incorporated into a survey-weighted multiple logistic regression analysis to examine the association between urinary PAHs and VI. The analysis was conducted using three models: Model 1, adjusted for age and sex; Model 2, included all variables from Model 1, plus race/ethnicity, education, and PIR; and Model 3, included all variables from Model 2, along with smoking status, alcohol consumption, BMI, physical activity, HEI, diabetes status, and hypertension status.

This study used supervised learning techniques to evaluate the association between PAHs and VI. Methods included Lasso, Elastic Net, Weighted Quantile Sum (WQS), and Bayesian Kernel Machine Regression (BKMR) to identify and interpret the roles of various PAHs in VI. Lasso regression selects key variables by penalizing the absolute values of regression coefficients, inducing sparsity and eliminating unimportant variables, thus simplifying the model and preventing overfitting. To optimize the penalty parameter λ, which balances model complexity and accuracy, we used the cv.glmnet function in R for cross-validation, identifying the optimal λ (lambda.min). To address multicollinearity, we applied Elastic Net regression, which combines Lasso and Ridge by applying L1 and L2 regularization to retain correlated predictor groups. Cross-validation was used to determine the optimal α (balancing Lasso and Ridge) and λ values, selecting the parameter setting that minimized prediction error. WQS regression assessed the overall impact of chemical mixtures on health outcomes by constructing a weighted index based on chemical concentration quantiles, effectively measuring total exposure to mixtures in relation to biological responses or health outcomes. The weight of each chemical reflects its importance to the outcome. We estimated weights through 10,000 bootstrap samples and validated the weighted index’s statistical significance using an independent test set. Additionally, BKMR was well-suited for capturing nonlinearity and interactions in exposure-response relationships. To improve computational efficiency, we standardized all continuous variables (e.g., PAH concentrations, age, HEI, BMI) and log-transformed PAH concentrations to address skewed distribution. We used the kmbayes function in R to perform 10,000 iterations, fitting the model and assessing the impact of PAH level changes from the 25th to 75th percentile on VI. To evaluate the indirect effects of PAH exposure on VI, we created diagrams showing cumulative effects of various PAHs and explored potential interactions among PAH metabolites using bivariate exposure-response plots. All models adjusted for confounders, including age, sex, race/ethnicity, education level, PIR, smoking status, alcohol consumption, BMI, physical activity, HEI, diabetes, and hypertension, to reduce bias and improve accuracy. Detailed information is available in Supplementary SM 1 [33,34,35,36,37,38,39].

We conducted a causal mediation analysis to explore the potential mediation effects between 2-Fluorene exposure and VI. A series of inflammatory markers were selected as mediators, including WBC, LYM, ALB, ALP, iron, and CRP. These markers may play a role in the biological pathways linking PAH exposure to VI. We constructed mediation and outcome models. In the mediation model, a generalized linear model was used to assess the relationship between 2-Fluorene exposure and each mediator to determine significant effects. In the outcome model, logistic regression assessed the direct and indirect effects of exposure and mediators on VI, while controlling for the direct effects of 2-Fluorene exposure. To estimate the indirect effects of 2-Fluorene on VI through different pathways, bootstrap sampling was used to infer the statistical significance of mediation effects, generating confidence intervals from 5000 samples. The mediation effect size was quantified by calculating the proportion of the total effect mediated (PM, Proportion Mediated). Given the complex relationships between PAH exposure, inflammation, and visual health, we constructed diagrams to illustrate these pathways. All analyses adjusted for potential confounders, including age, sex, race/ethnicity, education, PIR, smoking status, alcohol consumption, BMI, physical activity, HEI, diabetes, and hypertension. 

The analyses were performed using the R programming language (version 4.0.3, R Foundation for Statistical Computing) and the mediation package, generating *p*-values and confidence intervals for each mediation pathway. More detailed descriptions of these methods can be found in our previous publications [38,39].

## 3. Results

### 3.1. Characteristics of Participants

Participant demographics and health characteristics are summarized in Table 1. Participants had an average age of 50.07 years, with 50.22% being female. The majority of participants were non-Hispanic White (52.57%), followed by Hispanic (22.02%). In terms of education, 46.74% had an education beyond high school, and 28.29% had a high school diploma or less. Health data indicated that 72.65% of participants abstained from alcohol and 48.65% were nonsmokers. Diabetes and hypertension were prevalent in 13.69% and 32.55% of participants, respectively. VI was reported by 9.14% of participants. Urinary concentrations of PAHs were measured, with details provided in Table 1.

**Figure 2:**
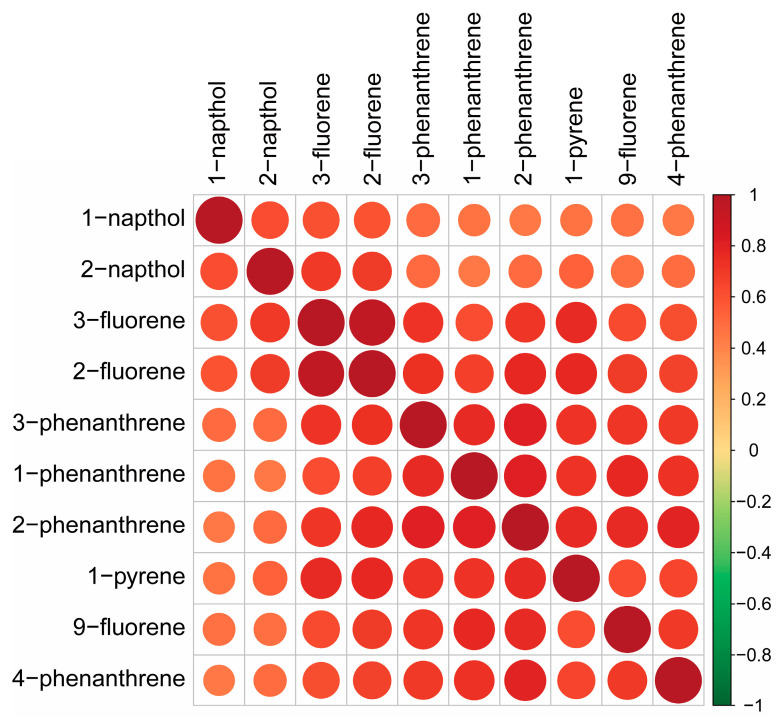
Correlation analysis of urinary concentrations for ten PAHs.

Figure 2 shows the urinary concentrations of ten PAHs, with correlation coefficients (ρ values) ranging from 0.44 to 0.96. The strongest correlation, with a ρ value of 0.96, was observed between urinary 3-fluorene and 2-fluorene. Additionally, urinary 2-phenanthrene showed strong correlations with several other PAHs: 0.78 with urinary 2-fluorene, 0.82 with urinary 1-phenanthrene, and 0.79 with urinary 4-phenanthrene.

### 3.2. Supervised Methods

#### 3.2.1. Lasso and Elastic Net

**Figure 3:**
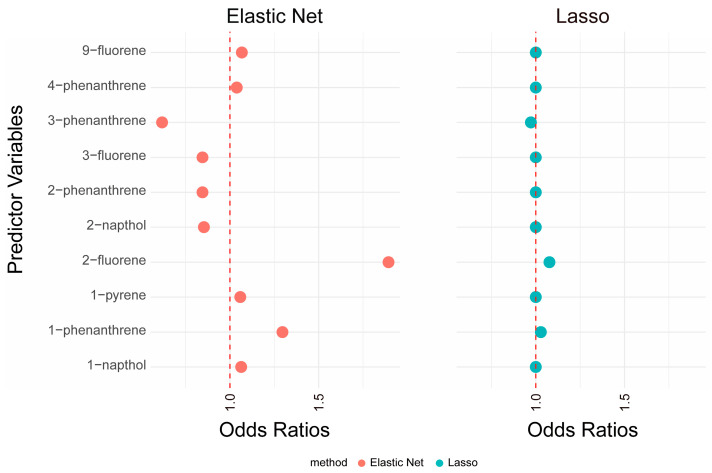
Coefficients of PAHs derived from variable selection models. The model adjusts for factors including age, sex, race/ethnicity, education level, PIR, smoking behavior, alcohol intake, BMI, physical activity levels, HEI, hypertension, and diabetes. All PAH concentrations were transformed using a logarithmic scale and normalized to nanograms per liter (ng/L).

In our study, the best λ value obtained from cross-validation for the LASSO regression model was 0.0039. Applying this λ to the Lasso model resulted in nonzero coefficients for two out of the ten PAHs assessed: 2-fluorene, with an odds ratio (*OR*) of 1.077, and 1-phenanthrene, with an *OR* of 1.028, as illustrated in Figure 3.

Similarly, for the elastic net model, cross-validation identified an optimal α of 0.3 and a λ of 1.53 × 10^−5^, closely aligning with the λ used in the lasso model. The elastic net approach yielded nonzero coefficients for six of the ten PAH variables analyzed, as shown in Figure 3. Notably, the same two variables, Lasso model-2-fluorene (*OR* = 1.89) and 1-phenanthrene (*OR* = 1.30), were selected, as were 9-fluorene (*OR* = 1.07) and 1-napthol (*OR* = 1.06). These results illustrate the consistent selection of key congeners across both models. However, the specific coefficients and their respective odds ratios varied slightly due to the different regularization parameters applied in the lasso and elastic net models.

#### 3.2.2. Weighted Quantile Sum Regression

**Figure 4:**
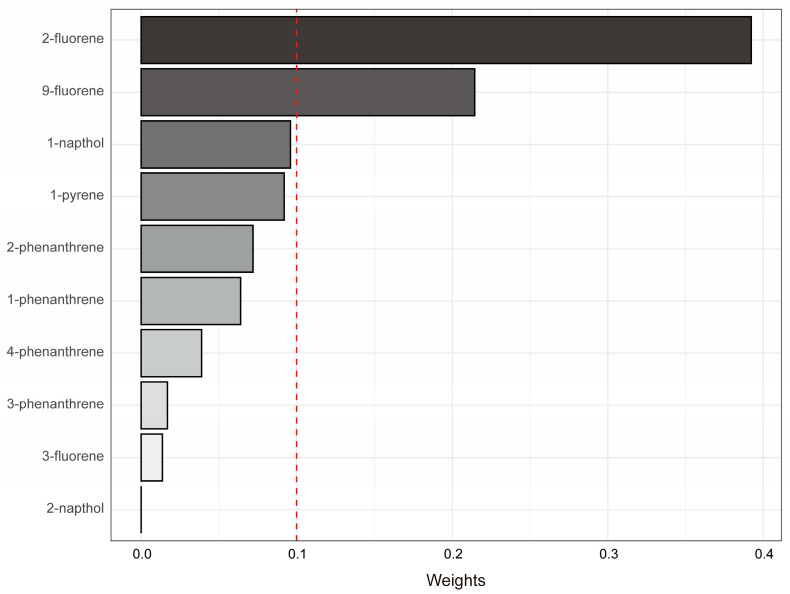
Variable weights estimated through the WQS method. The bar graph shows the weights assigned to ten PAHs. A dotted line at 0.1 marks the threshold for identifying key substances. The model accounts for variables such as age, sex, race/ethnicity, education level, PIR, smoking habits, alcohol consumption, BMI, physical activity, HEI, hypertension, and diabetes.

According to the WQS model, the coefficient for the mixture index was significantly positive (β = 0.35, 95% CI = 0.05 to 0.66, *p* = 0.02), indicating an association with VI. This significant result allows the weights of individual PAHs within the mixture to be seen as indicators of their relative importance. Notably, two PAHs—urinary 2-fluorene (weight = 0.39) and urinary 9-fluorene (weight = 0.21)—each had weights greater than 0.10, as shown in Figure 4. These substances emerged as the most significant components, together accounting for 60% of the overall weight of the mixture.

#### 3.2.3. Bayesian Kernel Machine Regression

**Figure 5:**
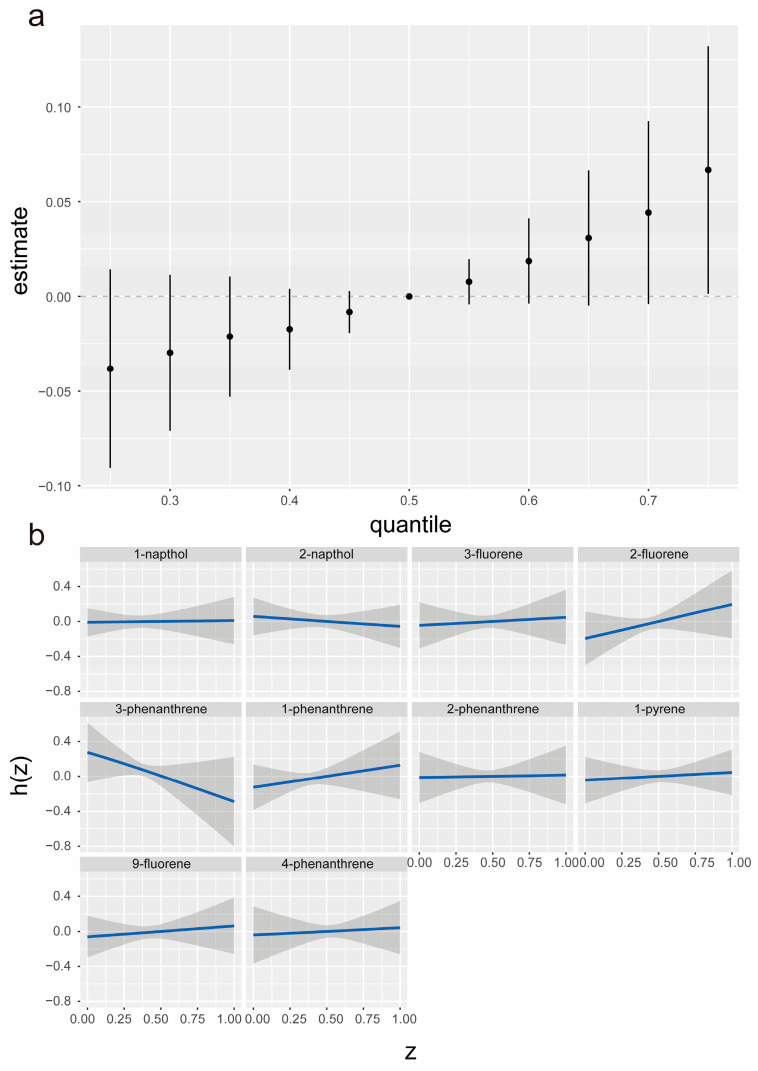
Estimation of the effects of PAH exposure on VI using the BKMR method. (**a**) The overall impact of PAH exposure on VI, with 95% confidence intervals, was evaluated by comparing different exposure percentiles to the median levels. (**b**) Associations for each PAH exposure with VI, along with 95% credible intervals, were estimated while keeping other exposures at their median levels. The model adjusted for age, sex, race/ethnicity, education, PIR, smoking behavior, alcohol intake, BMI, physical activity, HEI, hypertension, and diabetes. All PAH concentrations (ng/L) were log-transformed and standardized.

Figure 5 illustrates the association between VI and exposure to ten PAH metabolites, with VI treated as a binary outcome. The analysis indicated that the likelihood of VI increased with increasing exposure to PAH mixtures, showing a general upward trend. Importantly, this association was not consistently robust across all levels of exposure, with significant correlations observed at the highest concentrations (Figure 5a). Additionally, we assessed the individual contributions of each PAH metabolite to the likelihood of VI, while holding the levels of other metabolites at their median values. The results show positive correlations between the urinary concentrations of 2-fluorene, 1-phenanthrene, 9-fluorene, and 3-fluorene and VI, supported by positive posterior mean effect estimates (Figure 5b). However, the analysis revealed no significant interactions among these metabolites or with other metabolites examined in the study (Supplementary Figures S1 and S2).

#### 3.2.4. Mediation Analysis of the Association between 2-Fluorene Exposure and VI

**Figure 6:**
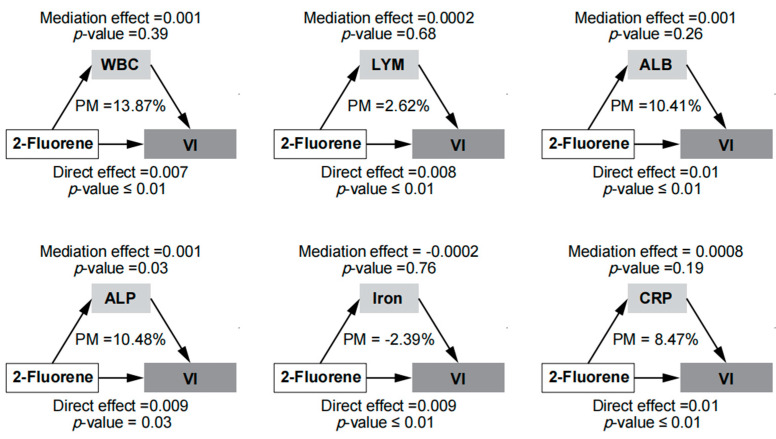
Mediation analysis of the association between 2-Fluorene exposure and VI. Abbreviations: WBC: White Blood Cell, LYM: Lymphocyte, ALB: Albumin, ALP: Alkaline Phosphatase, CRP: C-Reactive Protein, VI: Visual Impairment, PM: Proportion Mediated. The model adjusted for age, sex, race/ethnicity, education level, PIR, smoking habits, alcohol consumption, BMI, physical activity, HEI, hypertension, and diabetes.

Figure 6 illustrates the mediation analysis, highlighting the potential roles of various inflammatory markers in mediating the association between 2-Fluorene exposure and VI. WBC mediated 13.87% of the association between 2-Fluorene exposure and VI. However, the effect was not statistically significant (*p* = 0.39). LYM mediated a smaller proportion of 2.62%, which was also not statistically significant (*p* = 0.68). ALB mediated 10.41% of the association but did not reach statistical significance (*p* = 0.26). ALP showed a significant mediation effect (*p* = 0.03), mediating 10.48%, suggesting ALP may play a key biological role in the pathway between 2-Fluorene exposure and VI. Conversely, iron showed a negative mediation proportion (PM = −2.39%, *p* = 0.76) without statistical significance, while CRP mediated a positive proportion of 8.47%, which was also not significant (*p* = 0.19).

In the multivariable logistic regression analysis, total PAHs (∑PAHs) were summed and assessed for their association with VI. However, the results showed no statistically significant association between urinary PAHs and VI (Supplementary Table S2). Moreover, the trend test across ∑PAHs quartiles revealed no significant differences (*p* for trend = 0.61 in Model 3).

## 4. Discussion

In this study, we explored the complex relationship between PAHs and VI in American adults by applying four supervised machine learning techniques. Our findings indicate a significant association between PAH exposure and an increased incidence of VI, with specific compounds in the PAH mixture contributing to this effect. Notably, the lasso and elastic net models identified urinary 2-fluorene and urinary 1-phenanthrene as significant predictors of VI, underscoring their potential roles in the deterioration of visual health. Our findings were further supported by the WQS and BKMR analyses, which showed that elevated urinary levels of 2-fluorene and 9-fluorene are significantly associated with VI.

We further applied mediation analysis to explore how inflammatory markers mediate the relationship between urinary 2-Fluorene and VI. The results showed that ALP had a significant mediation effect, with a proportion of 10.48%, suggesting that ALP may play a key biological role in the pathway between 2-Fluorene exposure and VI. Results across all models consistently confirmed that urinary 2-Fluorene is a key factor negatively impacting visual health. This consistency validates the robustness of our findings and highlights the critical role of urinary 2-Fluorene in VI. Furthermore, the link between urinary 2-Fluorene and specific inflammatory markers suggests potential mechanisms by which PAH exposure may impact visual health through these biological pathways.

In environmental and occupational settings, human exposure to chemicals typically involves mixtures rather than single substances. This complexity poses challenges to traditional regression methods that focus on the impact of isolated factors. Machine learning methods are more adept at handling the complexities of multiple simultaneous exposures. Studies have shown that machine learning can better address nonlinear relationships and perform more effectively in scenarios with high dimensionality and multicollinearity than traditional methods such as logistic regression [40]. The machine learning methods are advantageous for managing large datasets and can uncover patterns and insights that traditional statistics might overlook. Increasing research supports these techniques, proving their effectiveness in modeling the health outcomes of mixed exposures in toxicological and environmental health studies.

Differences in identifying PAH congeners contributing to VI across statistical models primarily arise from variations in methodology and assumptions. Lasso and Elastic Net use regularization to select features, with Elastic Net able to retain groups of correlated variables, while Lasso may select only one. WQS regression assesses the contribution of each mixture component, while BKMR addresses nonlinearity and interaction effects. These methodological differences result in selecting different PAH congeners, but the results consistently show that urinary 2-Fluorene is the key contributor to VI. This consistency reinforces our confidence in urinary 2-Fluorene as a critical biomarker of health risk.

Our results identified a notable association between higher urinary PAH metabolite levels and VI. The mechanisms by which PAHs contribute to this impairment remain unclear. PAHs are metabolically activated to form reactive intermediates, such as epoxides and diols, which can covalently bond to cellular macromolecules, including proteins and DNA. This interaction disrupts cellular homeostasis and precipitates oxidative stress [41]. Oxidative stress is a critical determinant of the pathophysiology of vision-related disorders, particularly affecting cerebral regions tasked with visual processing and signal transduction. For example, empirical evidence links oxidative stress with retinopathies such as glaucoma and macular degeneration [42,43]. Consequently, it is postulated that chronic exposure to PAHs might increase susceptibility to these retinal pathologies. Prospective investigations are imperative to elucidate the specific impacts of PAHs on the visual system and to delineate their molecular mechanisms within ocular and neural matrices.

All four algorithms have consistently identified 2-fluorene as the most critical compound contributing to visual damage. Although no existing studies have directly linked 2-fluorene with visual disorders, the biological mechanisms by which it impacts vision remain undefined. The literature indicates a significant association between PAHs and inflammatory diseases [44,45]. For instance, Xu et al. highlighted the relationship between urinary biomarkers of PAHs in adolescents and liver function, noting that 2-fluorene is associated with a 3.56% increase in WBC, potentially indicating an impairment in liver function [44]. On the other hand, our results demonstrate no notable relationship between 2-fluorene and typical markers of inflammation, such as WBC, LYM, or levels of ALB, iron, and CRP. Nevertheless, a substantial association was detected between 2-fluorene and increased levels of ALP. ALP is an enzyme widely distributed across multiple human tissues, with notable concentrations in the liver, bones, and bile ducts. Previous research has indicated that ALP levels are also associated with auditory impairments [46]. Moreover, ALP is crucial in phosphatase metabolism, affecting cellular energy production and utilization, and acting as an indicator of chronic inflammation. 2-Fluorene is a common PAH, primarily originating from the incomplete combustion of organic matter [47]. Common exposure pathways include motor vehicle exhaust, industrial emissions, tobacco smoke, and the consumption of grilled or barbecued foods. Although we found no significant association between 2-fluorene and conventional inflammatory markers (e.g., WBC and LYM), its correlation with elevated ALP levels suggests a potential role in metabolic regulation. Specifically, it may indirectly contribute to VI by disrupting the metabolic environment in the visual nervous system. Based on these findings, we recommend that risk managers increase monitoring of key PAH compounds, such as 2-fluorene, particularly in high-exposure environments. We also suggest further research to explore how PAHs impact visual health through metabolic pathways, particularly those involving ALP. For individuals with existing VI, we recommend regular testing of 2-fluorene levels to better understand its link to visual damage, providing valuable insights for future health management and prevention. 

Our study offers significant advantages. Using a comprehensive population dataset, this study methodically evaluated the effects of both single and combined PAHs on biomarkers linked to VI. By employing four supervised machine learning algorithms, this research identifies significant compounds within complex mixtures and assesses their aggregate impact on visual outcomes. The application of four supervised learning models elucidates the association between PAH exposure and VI, while revealing potential interactive effects. Crucially, this study identified 2-fluorene as a compound of considerable interest due to its strong association with VI, setting the stage for future mechanistic explorations. Furthermore, our analysis considered the impact of diet quality and physical activity on vision loss, enhancing the credibility of our results. However, the intricacy of our models imposes certain restrictions. For example, determining the distinct effects of individual compounds can be difficult when two exposures are highly related. Notwithstanding these obstacles, our findings indicate that certain compounds may exert a more substantial influence even under high correlations, emphasizing the significance of comprehending the combined impact of PAHs on well-being. Notably, the WQS framework assigns a lower weight to 9-fluorene compared to the BKMR model, which may result from methodological constraints inherent to the WQS approach. When two exposures are strongly correlated and one is given a minimal weight, the model may adjust the other exposure’s weight to account for their correlation.

The limitations of this study require thoughtful evaluation. The cross-sectional design limits the ability to establish causal relationships between PAH exposure and markers of VI. To address this limitation, future research should employ longitudinal designs and in vivo experiments to substantiate and elucidate the underlying causative pathways. Additionally, the representativeness of our findings is potentially compromised by the uniform demographics of our sample, which are limited to American adults. Furthermore, it is essential to conduct similar studies in regions with diverse environmental exposures and demographic profiles to generalize the findings and gain a more comprehensive understanding of the global relationship between PAH exposure and VI. This approach would also support region-specific recommendations for PAH exposure monitoring and mitigation, particularly in identifying key compounds like 2-fluorene, which may pose greater risks in specific populations or environments, thereby guiding targeted public health interventions. 

Moreover, the reliance on single-point PAH concentration data calls for cautious interpretation, given the potential for exposure fluctuations. Therefore, future studies should consider repeated measures of exposure over time to better capture the dynamic nature of PAH exposure and its health effects. Additionally, the data for this study were obtained from a public database that, while providing essential toxicant exposure information, lacks variables on environmental data, exposure pathways, occupational activities, and industrialized areas. As a result, we are unable to assess the potential impact of these factors on the relationship between PAH exposure and visual health outcomes. Thus, future studies should incorporate these environmental factors to better evaluate the impact of PAH exposure on population health in various occupational or industrial contexts. Although we adjusted for several confounders, latent factors like genetic variability and long-term environmental interactions could affect PAH pharmacokinetics, potentially altering urinary metabolite concentrations and their association with VI. One limitation of our study is the lack of directed acyclic graphs (DAGs) or other formal causal inference tools to systematically identify confounders. We selected potential confounders based on existing literature and known associations between these variables, PAH exposure, and visual health outcomes. Tennant et al. emphasized the importance of systematic methods like DAGs for identifying confounders [48]. Future research could benefit from using DAGs or similar tools to better account for both measured and latent confounders, thereby improving causal inference and reducing bias from uncontrolled confounding. Finally, in line with these limitations, future research should also focus on translating findings into actionable public health recommendations, such as developing guidelines for PAH exposure reduction and more stringent monitoring in high-risk areas.

## 5. Conclusions

In this study, the results revealed a significant association between PAH exposure and VI and identified specific PAH types with exposure–response relationships, highlighting key pollutants likely contributing to the observed health effects. These findings suggest that PAHs may negatively impact human health. However, further research is needed to better understand the mechanisms linking urinary PAH metabolites and VI.

## Figures and Tables

**Table 1:** Baseline characteristics of the study participants (*n* = 1149) in the NHANES 2003–2004 survey.

Characteristic	Overall
Age (years), mean, and sd	50.07 ± 19.05
Gender, % and *n*	
Male	572 (49.78%)
Female	577 (50.22%)
Ethnicity, % and *n*	
Hispanic	253 (22.02%)
Non-Hispanic White	604 (52.57%)
Non-Hispanic Black	238 (20.71%)
Other race	54 (4.70%)
Education, % and *n*	
Less than high school	325 (28.29%)
High school	287 (24.98%)
More than high school	537 (46.74%)
Poverty index, % and *n*	
≤1	203 (17.67%)
>1	874 (76.07%)
missing	72 (6.27%)
Smoking, % and *n*	
Non-smoker	559 (48.65%)
Ever smoker	307 (26.72%)
Current smoking	282 (24.54%)
missing	1 (0.09%)
Alcohol consumption, % and *n*	
Non-drinker	821 (71.45%)
Moderate	99 (8.62%)
Heavy	160 (13.93%)
Missing	69 (6.01%)
Diabetes, % and *n*	
No	1003 (87.29%)
Yes	128 (11.14%)
Missing	18 (1.57%)
Hypertension, % and *n*	
No	762 (66.32%)
Yes	374 (32.55%)
Missing	13 (1.13%)
HEI Index, mean and sd	48.54 ± 12.51
Physical Activity, MET-min/wk, No. (%)	
<600	536 (46.65%)
600 ≤, <1200	178 (15.49%)
≥1200	435 (37.86%)
BMI Category (kg/m^2^), % and n	
<25	354 (30.81%)
25–30	385 (33.51%)
≥30	401 (34.90%)
Missing	9 (0.78%)
Visual impairment, % and *n*	
No	1044 (90.86%)
Yes	105 (9.14%)
Urinary 1-napthol, ng/L	2621.40 (1125.70–9253.40)
Urinary 2-napthol, ng/L	3133.80 (1340.00–8616.20)
Urinary 3-fluorene, ng/L	97.40 (44.90–340.20)
Urinary 2-fluorene, ng/L	279.2 (130.40–778.30)
Urinary 3-phenanthrene, ng/L	113.40 (55.90–216.40)
Urinary 1-phenanthrene, ng/L	160.6 (83.20–282.50)
Urinary 2-phenanthrene, ng/L	63.60 (30.50–122.60)
Urinary 1-pyrene, ng/L	79.50 (35.60–175.00)
Urinary 9-fluorene, ng/L	282.70 (139.60–565.80)
Urinary 4-phenanthrene, ng/L	25.30 (11.3–53.70)

## Data Availability

The data presented in this study are available in the National Center for Health Statistics (NCHS) repository at https://www.cdc.gov/nchs/nhanes/ (accessed 1 May 2024). These data were derived from the following resource available in the public domain: NHANES. For detailed information about the vision examination protocol used during the NHANES 2003–2004, please visit https://wwwn.cdc.gov/Nchs/Nhanes/2003-2004/VIX_C.htm (accessed 1 May 2024).

## Supplementary Materials

The following supporting information can be downloaded at: https://www.mdpi.com/article/10.3390/toxics12110789/s1, Table S1: Detection rates of various PAHs in urine samples; Table S2: Association between total PAHs and visual impairment in the survey-weighted multivariable logistic regression model; Figure S1: Bivariate exposure-response functions for PAHs; Figure S2: Interaction terms for individual PAHs were evaluated using the BKMR method; SM 1: Overview of machine learning methods and their application to PAH-VI analysis.
