# Supplementary material for: Using Four Machine Learning Methods to Analyze the Association Between Polycyclic Aromatic Hydrocarbons and Visual Impairment in American Adults: Evidence from NHANES
Source: Toxics. 2024 Oct 29;12(11):789. doi: 10.3390/toxics12110789 (PMC11598647; doi:10.3390/toxics12110789)
Supplement: Supplementary file 1 [file toxics-12-00789-s001.zip › toxics-3194788-supplementary.pdf]

Supplementary Material

Using Four Machine Learning Methods to Analyze the Association between Polycyclic Aromatic Hydrocarbons and Visual Impairment in American Adults: Evidence from NHANES

Xiaowei Zang, Wei Zhou, Hengguo Zhang and Xiaodong Zang

Table S1. Detection rates of various PAHs in urine samples.

Table S2. Association between total PAHs and visual impairment in the survey-weighted multivariable logistic regression model.

Figure S1. Bivariate exposure-response functions for PAHs.

Figure S2. Interaction terms for individual PAHs were evaluated using the BKMR method.

SM 1: Overview of machine learning methods and their application to PAH-VI analysis.

Table S1. Detection rates of various PAHs in urine samples.

Chemical	Sample size(*n*)		
	At or above the detection limit (*N*)	Below lower detection limit (*N*)	Detection rate (%)
Urinary 1-napthol	2593	2	99.92
Urinary 2-napthol	2575	0	100
Urinary 3-fluorene	2496	6	99.76
Urinary 2-fluorene	2521	0	100
Urinary 3-phenanthrene	2420	6	99.75
Urinary 1-phenanthrene	2495	1	99.96
Urinary 2-phenanthrene	2481	31	98.77
Urinary 1-pyrene	2499	16	99.36
Urinary 9-fluorene	2504	0	100
Urinary 4-phenanthrene	2230	213	91.28

Table S2. Association between total PAHs and visual impairment in the survey-weighted multivariable logistic regression model.

Exposure	Model 1	*p*	Model 2	*p*	Model 3	*p*
	OR (95% CI)		*OR* (95% CI)		*OR* (95% CI)	
∑ PAHs,ng/L	1.00(1.00, 1.00)	0.13	1.00(1.00, 1.00)	0.06	1.00(1.00, 1.00)	0.05
∑PAHs (quartiles)						
Q1	1 (reference)		1 (reference)		1 (reference)	
Q2	1.24(0.70, 2.18)	0.46	1.24(0.69, 2.21)	0.47	1.23(0.68, 2.21)	0.49
Q3	1.11(0.62, 1.99)	0.73	1.01(0.55, 1.85)	0.97	0.99(0.53, 1.82)	0.96
Q4	1.02(0.56, 1.86)	0.94	0.92(0.46, 1.85)	0.81	0.87(0.42, 1.77)	0.69
p for trend	0.97		0.72		0.61	

Model 1: adjusted for age and sex. Model 2 included all variables from Model 1 plus race/ethnicity, education, PIR. Model 3 included all variables from Model 2 plus smoking status, alcohol consumption, BMI, physical activity, HEI, diabetes status, and hypertension status.

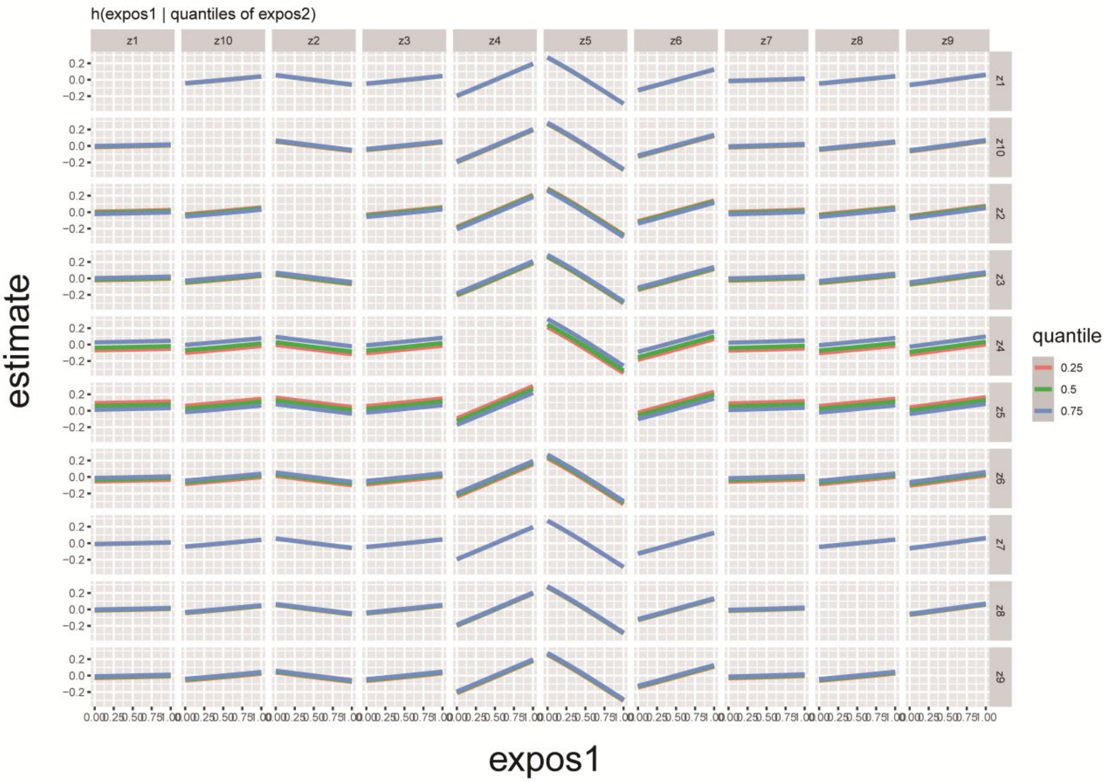

Figure S1. Bivariate exposure-response functions for PAHs. Bivariate exposure-response functions for PAHs were evaluated with the concentration of one PAH set at the 25th, 50th, or 75th percentile, while keeping all other PAH concentrations at their median values. The model adjusted for covariates such as age, sex, race/ethnicity, education level, PIR, smoking behavior, alcohol intake, BMI, physical activity, HEI, hypertension, and diabetes. PAH levels were log-transformed and standardized. The PAHs analyzed included 1-naphthol (z1), 2-naphthol (z2), 3-fluorene (z3), 2-fluorene (z4), 3-phenanthrene (z5), 1-phenanthrene (z6), 2-phenanthrene (z7), 1-pyrene (z8), 9-fluorene (z9), and 4-phenanthrene (z10).

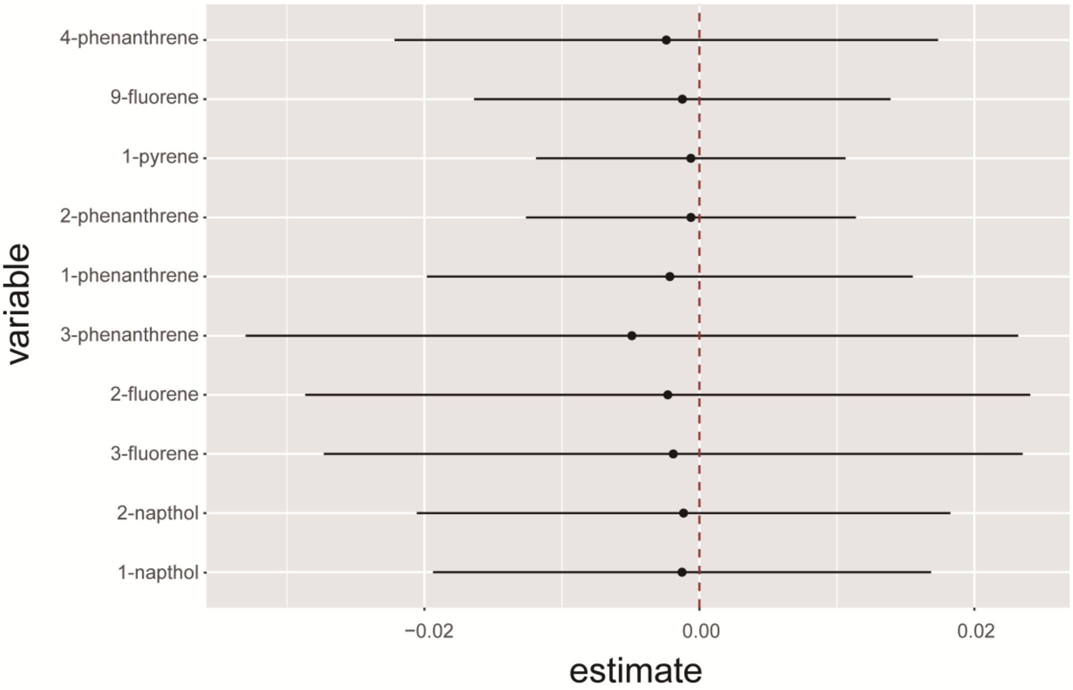

Figure S2. Interaction terms for individual PAHs were evaluated using the BKMR method. Each point reflects how the effect size of a specific PAH changes when other PAH concentrations are at the 75th versus the 25th percentile, with 95% credible intervals. The model accounted for variables such as age, sex, race/ethnicity, education, PIR, smoking status, alcohol consumption, BMI, physical activity, HEI, hypertension, and diabetes. PAH levels were log-transformed and standardized.

SM 1: Overview of machine learning methods and their application to PAH-VI analysis.

Our research employed supervised learning methods, including variable selection techniques (Lasso and Elastic Net), weighted quantile sum regression (WQS), and Bayesian kernel machine regression (BKMR), to assess the relationship between PAHs and visual impairment (VI). Initially, we applied Lasso regression, which promotes model sparsity by penalizing the absolute values of the coefficients, resulting in some being reduced to zero [1,2]. This approach aids in variable selection and model simplification, enhancing interpretability while preventing overfitting. We optimized the penalty parameter, λ, using the cv.glmnet function from the glmnet package in R, which performs cross-validation by varying λ on an exponential scale. The analysis began by setting a random seed for reproducibility and defining a range of λ values from 0.5^{-20} to 0.5^{20}. A selective penalty application ensured that only PAH variable coefficients were penalized, allowing the inclusion of non-PAH covariates as control variables. We identified the optimal λ, known as lambda.min, which minimized cross-validation error, thus balancing model complexity and accuracy.

Additionally, to address multicollinearity among predictors, we used elastic net regression, which combines the properties of Lasso and Ridge regressions. Elastic net applies the L1 penalty from Lasso and the L2 penalty from Ridge for coefficient shrinkage, enabling the retention of correlated predictors as a group and reducing Lasso's tendency to select only one variable from highly correlated groups [3]. We adjusted both the α parameter, which balances the influence of Lasso and Ridge (ranging from 0.1, nearly pure Ridge, to 1, pure Lasso), and λ for overall penalty strength. The optimal values for α and λ were determined through cross-validation with cv.glmnet, and values that minimized prediction error were selected. These optimal settings were used to identify significant PAH variables, indicated by nonzero coefficients in the final model.

The WQS methodology assesses how chemical mixtures collectively influence outcomes, including biological responses and health effects [4]. Using the gWQS package in R, this model creates a weighted index based on the quantiles of chemical concentrations, representing the overall exposure level of the mixture. The weight of each chemical in the index reflects its significance. The process begins with bootstrap sampling from the training dataset, followed

by weight estimation for each chemical using a nonlinear numerical optimization algorithm. The model was executed with 10,000 bootstrap samples. The final weighted index, computed as a weighted average of the bootstrap samples, was validated using an independent test set. Weights were adjusted based on their relevance to the outcome, as indicated by the regression beta coefficients. A subset comprising 60% of our samples was used to evaluate the index's statistical significance and predictive power. A threshold of 0.10 is set to identify significant chemicals, although this threshold is indicative and subject to weight variability and explained variance considerations. A positive directional constraint is applied to include only chemicals that increase risk, thereby improving the interpretability of the index.

In addition, this study employed BKMR to explore the relationship between exposure to ten PAHs and VI, which was defined as a binary outcome. This statistical approach is particularly suited for modeling nonlinear and interactive exposure–response relationships and utilizes a Gaussian kernel to capture complex interactions among exposures[3-5]. To enhance computational efficiency and optimize model fitting, all continuous variables, such as PAHs, age, healthy eating index, and BMI, were normalized. A log transformation was applied to PAHs to correct for skewness, while other continuous variables were standardized by centering and scaling. Categorical variables were converted into dummy variables. To ensure the stability and reproducibility of the results, a random seed was set, and 100 knots were generated using the cover.design() function from the fields package. The kmbayes function from the 'bkmr' R package was used, running 10,000 iterations with the family parameter set to 'binomial' for the binary outcome. The analysis assessed the effects of PAH levels between the 25th and 75th percentiles while keeping other PAHs fixed at the 25th, 50th, or 75th percentiles to evaluate both individual and combined effects. Additionally, cumulative effects of the ten PAH metabolites were plotted, and bivariate exposure-response relationships between PAH pairs were visualized with other PAHs held at their median values. This analysis was crucial in investigating potential interactions among PAH metabolites, offering valuable insights into how combined exposures may impact VI. All statistical analyses were conducted using R (version 4.0.3, R Foundation for Statistical Computing). More detailed descriptions of these methods can be found in our previous publications [6,7].

References

1. Sumner, S.A.; Bowen, D.; Holland, K.; Zwald, M.L.; Vivolo-Kantor, A.; Guy, G.P., Jr.; Heuett, W.J.; Pressley, D.P.; Jones, C.M. Estimating Weekly National Opioid Overdose Deaths in Near Real Time Using Multiple Proxy Data Sources. *JAMA Netw Open* **2022**, *5*, e2223033, doi:10.1001/jamanetworkopen.2022.23033.
2. Ramos, K.J.; Hee Wai, T.; Stephenson, A.L.; Sykes, J.; Stanojevic, S.; Rodriguez, P.J.; Bansal, A.; Mayer-Hamblett, N.; Goss, C.H.; Kapnadak, S.G. Development and Internal Validation of a Prognostic Model of the Probability of Death or Lung Transplantation Within 2 Years for Patients With Cystic Fibrosis and FEV(1) ≤ 50% Predicted. *Chest* **2022**, *162*, 757-767, doi:10.1016/j.chest.2022.05.021.
3. Lu, Y.; Zhang, Y.; Guan, Q.; Xu, L.; Zhao, S.; Duan, J.; Wang, Y.; Xia, Y.; Xu, Q. Exposure to multiple trace elements and miscarriage during early pregnancy: A mixtures approach. *Environ Int* **2022**, *162*, 107161, doi:10.1016/j.envint.2022.107161.
4. Huang, Q.; Wan, J.; Nan, W.; Li, S.; He, B.; Peng, Z. Association between manganese exposure in heavy metals mixtures and the prevalence of sarcopenia in US adults from NHANES 2011-2018. *J Hazard Mater* **2024**, *464*, 133005, doi:10.1016/j.jhazmat.2023.133005.
5. Bobb, J.F.; Valeri, L.; Claus Henn, B.; Christiani, D.C.; Wright, R.O.; Mazumdar, M.; Godleski, J.J.; Coull, B.A. Bayesian kernel machine regression for estimating the health effects of multi-pollutant mixtures. *Biostatistics* **2015**, *16*, 493-508, doi:10.1093/biostatistics/kxu058.
6. Zang, X.; Qin, W.; Xiong, Y.; Xu, A.; Huang, H.; Fang, T.; Zang, X.; Chen, M. Using three statistical methods to analyze the association between aldehyde exposure and markers of inflammation and oxidative stress. *Environ Sci Pollut Res Int* **2023**, *30*, 79437-79450, doi:10.1007/s11356-023-27717-4.
7. Zang, X.; Feng, L.; Qin, W.; Wang, W.; Zang, X. Using machine learning methods to analyze the association between urinary polycyclic aromatic hydrocarbons and chronic bowel disorders in American adults. *Chemosphere* **2024**, *346*, 140602, doi:10.1016/j.chemosphere.2023.140602.
